# Effects of Deformed Wing Virus Infection on Expressions of Immune- and Apoptosis-Related Genes in Western Honeybees (*Apis mellifera*)

**DOI:** 10.3390/insects12010082

**Published:** 2021-01-19

**Authors:** Wannapha Mookhploy, Sasiprapa Krongdang, Panuwan Chantawannakul

**Affiliations:** 1 Bee Protection Laboratory, Department of Biology, Faculty of Science, Chiang Mai University, Chiang Mai 50200, Thailand; wannapha.mookhploy@gmail.com or; 2 Graduate School, Chiang Mai University, Chiang Mai 50200, Thailand; 3 Faculty of Science and Social Sciences, Burapha University Sa Kaeo Campus, Sa Kaeo 27160, Thailand; sasiprapakr@gmail.com or; 4 Environmental Science Research Center, Faculty of Science, Chiang Mai University, Chiang Mai 50200, Thailand

**Keywords:** *Apis mellifera*, apoptosis, deformed wing virus, host defense, immune, bee mortality

## Abstract

**Simple Summary:**

Deformed wing virus (DWV) is a widespread viral pathogen in western honeybees. This work investigated the effects of DWV on honeybee and host defense by analyzing the immune and apoptosis gene expressions. The results revealed that the mortality rates and deformed wing symptoms in newly emerged adult bees increased at high initial concentration of DWV injection. Moreover, abnormality of wings in DWV injected bees did not correlate with the virus levels nor the immune and apoptosis responses. After DWV injection, the immune response was activated in pupae and newly emerged adult bees. However, apoptosis genes were stimulated only in newly emerged adult bees but suppressed in honeybee pupae. The apoptosis gene suppression during the initial phase of infection may promote the DWV survival and parasitism in the honeybee host.

**Abstract:**

Honeybees are globally threatened by several pathogens, especially deformed wing virus (DWV), as the presence of DWV in western honeybees is indicative of colony loss. The high mortality rate is further exacerbated by the lack of effective treatment, and therefore understanding the immune and apoptosis responses could pave an avenue for the treatment method. In this study, DWV was directly injected into the white-eyed pupae stage of western honeybees (*Apis mellifera*). The DWV loads and selected gene responses were monitored using the real-time PCR technique. The results showed that honeybee pupae that were injected with the highest concentration of viral loads showed a significantly higher mortality rate than the control groups. Deformed wings could be observed in newly emerged adult bees when the infected bees harbored high levels of viral loads. However, the numbers of viral loads in both normal and crippled wing groups were not significantly different. DWV-injected honeybee pupae with 10^4^ and 10^7^ copy numbers per bee groups showed similar viral loads after 48 h until newly emerged adult bees. Levels of gene expression including immune genes (*defensin, abaecin*, and *hymenoptaecin*) and apoptosis genes (*buffy, p53, Apaf1, caspase3-like, caspase8-like,* and *caspase9-like*) were analyzed after DWV infection. The expressions of immune and apoptosis genes were significantly different in infected bees compared to those of the control groups. In the pupae stage, the immune genes were activated by injecting DWV (*defensin* and *hymenoptaecin*) or *Escherichia coli* (*defensin, abaecin,* and *hymenoptaecin*), a positive control. On the contrary, the expression of apoptosis-related genes (*buffy*, *caspase3-like*, *caspase8-like*, and *caspase9-like* genes) was suppressed at 96 h post-infection. In DWV-infected newly emerged adult bees, *abaecin, hymenoptaecin, Apaf1*, and *caspase8-like* genes were upregulated. However, these genes were not significantly different between the normal and crippled wing bees. Our results suggested that DWV could activate the humoral immunity in honeybees and that honeybee hosts may be able to protect themselves from the virus infection through immune responses. Apoptosis gene expressions were upregulated in newly emerged adult bees by the virus, however, they were downregulated during the initial phase of viral infection.

## 1. Introduction

Honeybees are invaded by several pathogens such as bacteria, fungi, parasites, and viruses, and therefore have developed several defense mechanisms against parasites and pathogens [1]. One of the mechanisms is the behavioral defense such as grooming as well as hygienic and necrophoric behaviors that reduce the pathogen loads in the colony [1,2,3]. Physical barriers (insect cuticle and epithelial layers) are the first defense against pathogen invasion into the body [1]. Moreover, a chemical barrier in the insect gut can inhibit foreign pathogens. The second line of defense, namely, cellular and humoral immune systems, is activated in response to pathogens that overcome these barriers [4]. The immune systems deploy pattern recognition receptors (PRRs) that bind to the pathogen-associated molecular patterns (PAMPs) on the surface of pathogens (viral double-stranded RNA (dsRNA), bacterial peptidoglycans, and fungal β-glucans). The binding between PAMP and PRRs activates the hemocyte-mediated cellular immune response through many signaling pathways including Toll, Imd, Jak-STAT, and JNK. The activation of these pathways induces several processes such as phagocytosis, nodule formation, encapsulation mechanisms, and the synthesis of antimicrobial peptides (AMP) [5].

Deformed wing virus (DWV) is one of honeybee pathogens that causes weakening and can eventually lead to the death of honeybees [6,7]. Transmitted via horizontal and vertical routes, this virus is associated with mite infestation and colony collapse [8,9,10]. Parasitic mites including *Varroa* and *Tropilaelaps* are common vectors of DWV in honeybees [11,12]. In a colony that showed no mite infestation, low titer of DWV was detected whereas mite-infested colonies displayed increased levels of DWV titer [13]. DWV-infected honeybees may show characteristics clinical symptoms including deformed wings, shortened abdomens, discoloring, and behavioral abnormalities. However, not all honeybees with DWV infection display the symptoms of the disease [14].

Apoptosis or programmed cell death is an important component of various processes including normal cell development, embryonic development, function of the immune system, hormone-dependent atrophy, and chemical-induced cell death [15,16]. Many lines of evidence have demonstrated that viral infection induces apoptosis in insects and that the infection is mitigated by the elimination of the infected cells [17,18,19]. The mitochondrial pathway of apoptosis is induced by cytochrome *c* binding to Apaf-1, an event that stimulates the activation of caspases [20]. Blocking the release of cytochrome *c* halts apoptosis, and can be achieved by the activation of *buffy,* a cell death inhibitor functionally similar to the pro-survival Bcl-2 family of proteins in mammals [21]. Apoptosis is also activated by several genes including, *apaf1*, *p53*, and *caspase*, which induces caspases that are the final proteases that activate apoptosis [22]. A study by Koziy et al. suggested that apoptosis occurred in the hypopharyngeal gland cells of DWV-infected newly emerged honeybees [23]. Although immune and apoptosis-related gene responses to the virus were investigated in several studies [5,24,25,26], information on the temporal relationship between DWV levels and these genes is still scarce.

This study aimed to demonstrate the effects of DWV on immune- and apoptosis-related gene responses in western honeybees (*Apis mellifera*). Antimicrobial peptide (AMP) genes were tested, including, *defensin, abaecin,* and *hymenoptaecin*. Moreover, six apoptosis-related genes, namely, *buffy*, *p53*, *Apaf1*, *caspase3-like*, *caspase8-like*, and *caspase9-like*, were also investigated. The results of this study would provide a better understanding of DWV survival in honeybees. It may be helpful to discover ways of virus elimination in the future.

## 2. Materials and Methods

### 2.1. Honeybee Samples

Crippled honeybees were collected from the Bee Protection Laboratory (BeeP) located within Chiang Mai University, Suthep Subdistrict, Mueang Chiang Mai District, Chiang Mai, Thailand (18°48′14.3″ N 98°57′22.2″ E). All honeybee pupae with no disease symptoms and mite infection were obtained from 3 healthy colonies located in the Bee Protection center, Suthep Subdistrict, Mueang Chiang Mai District, Chiang Mai, Thailand, during 2016–2017.

### 2.2. Bacterial Strains and Media

The *Escherichia coli* ATCC 25922 was used in this study. *E. coli* was cultured in nutrient broth (NB) and incubated at 37 °C until grown to an absorbance of A_550_ = 0.5 (~3 × 10^8^ cells/mL) [27,28]. The suspension was centrifuged at 2720× *g* for 5 min to collect cells (K3 Series, Centurion Scientific Ltd., London, UK). The cells were washed twice and adjusted to the desired concentration with phosphate-buffered saline (PBS) [28].

### 2.3. Preparation of DWV Lysate

Five individual honeybees with apparent symptoms of DWV infection were frozen in liquid nitrogen and crushed with a mortar and pestle. The ground bees were suspended in 5 mL of phosphate buffer solution (pH 7.4). The supernatant was collected after centrifugation at 6440× *g* for 10 min at 4 °C (K3 Series, Centurion Scientific Ltd., London, UK). The DWV lysate was filtered through a 0.2 micron filter to eliminate bacteria, fungi, and nosema [29]. DWV lysate was subject to quantitative real-time PCR (RT-PCR) to confirm the absence of other bee viruses (acute bee paralysis virus (ABPV) [30], black queen cell virus (BQCV) [30], chronic bee paralysis virus (CBPV) [30], Israeli acute paralysis virus (IAPV) [31], Kashmir bee virus (KBV) [30], and sacbrood virus (SBV) [30]) in the lysate (see virus primer sequence in Supplementary Materials Table S1). Lysate without all 6 honeybee viruses was used for this study (Table S2). Moreover, the DWV genotype was confirmed by RT-PCR (DWV-A, forward: 5′-CGTCGGCCTATCAAAG-3′; reverse: 5′-CTTTTCTAATTCAACTTCACC-3′ and DWV-B, forward: 5′-GCCCTGTTCAAGAACATG-3′; reverse: 5′-CTTTTCTAATTCAACTTCACC-3′) as previously described [32]. The lysate was kept at −80 °C until use [33].

### 2.4. In Vitro Challenge of Honeybee Pupae with Virus and Bacteria 

White-eyed honeybee pupae were collected for this experiment. In the first part of the experiment, honeybee pupae were randomly separated into 7 groups. The control group was untreated (*n* = 90, 30 bees per treatment performed in triplicate). In experimental groups, honeybee pupae were injected laterally between the second and third tergite of the abdomen with 2 µL of PBS per bee (a negative control group), *Escherichia coli* (a positive control group), and 4 dilutions of number of DWV genome copies (10^4^, 10^5^, 10^6^, and 10^7^ copy numbers per bee) (*n* = 90 for each group, 30 bees per treatment performed in triplicate). Injected honeybee pupae were incubated at 34 ± 1 °C and 70% relative humidity until they developed into newly emerged adult bees [27,28]. The surviving honeybee pupae were recorded every day until the end of the experiment. Moreover, DWV levels and expressions of immune- and apoptosis-related genes in newly emerged adult bees were analyzed. The second part of the experiment investigated the effects of DWV levels on the immune- and apoptosis-related genes during the pupal development. The white-eyed honeybee pupae were separated into 5 groups: control (un-injected), PBS, *E. coli*, and 2 infected pupae groups injected with DWV (10^4^ and 10^7^ copy numbers per bee). The honeybee pupae from each treatment group (*n* = 60, 20 bees per treatment performed in triplicate) were randomly collected at 0, 6, 12, 24, 48, 72, and 96 h post-treatment and immediately put in liquid nitrogen. All samples were stored at −80 °C for further analyses.

### 2.5. RNA Extraction and cDNA Synthesis

Total RNA of pupae and newly emerged adult bees were individually extracted using TRIzol (Invitrogen, Carlsbad, CA, USA) following the manufacturer’s protocol. The RNA quantity was determined by a BioDrop Duo spectrophotometer (BioDrop Ltd., Cambridge, UK). The first-strand complementary DNA (cDNA) was then synthesized using the Tetro cDNA synthesis kit following the manufacturer’s protocol (Bioline, Alexandria, NSW, Australia).

### 2.6. Quantitative Real-Time PCR Parameters

The number of DWV genome copies was analyzed using SensiFAST SYBR No-ROX Kit master mix (Bioline, Alexandria, NSW, Australia). The DWV titers of samples were determined by the absolute quantification method. The standard curve was established using 7 concentrations of 10-fold dilutions of DWV insert in TOPO TA Cloning plasmid (Invitrogen, Carlsbad CA, USA). The total RT-qPCR reaction volume was 20 μL, composed of 10 µL of 2× SensiFAST SYBR No-ROX Mix, 0.8 µL of each 10 µM primer, 1 μL (≈100 ng) of cDNA template, and 7.4 µL of H_2_O. The reaction was performed in the BioRad iCycler iQ 5 (Bio-Rad Crop., Hercules, CA, USA). Amplification was performed as follows: samples were held at 50 °C for 30 min and 95 °C for 10 min, followed by 40 cycles of 95 °C for 30 s, 55 °C for 1 min, and 72 °C for 30 s. The melting curve was obtained by incubating the PCR products for 1 min at 95 °C, ramping down to 55 °C at a rate of 0.2 °C/s. The dissociation curve was constructed using 81 complete cycles of incubation from 55 °C to 95 °C with 0.5 °C/s increment. Relative quantification in RT-PCR was determined for AMPs and apoptosis-related genes. Ribosomal protein subunit 5 (RPS5) and *β*-actin were used as reference genes. All primers are shown in Table 1. All reactions were performed using a thermal program of 95 °C for 30 s followed by 40 cycles of 95 °C for 30 s, 60 °C for 30 s, and 72 °C for 1 min and 30 s. The final RT-qPCR amplicon was confirmed by the analysis of the melting curve by increasing the temperature from 55 °C to 95 °C with 0.5 °C/s increment. Negative controls (no template) were included in each run. All reactions were performed in triplicate. Gene expression was calculated as 2^−∆∆CT^ [34].

### 2.7. Data Analyses

Statistical analyses were performed using IBM SPSS Statistics Version 25.0 (IBM Corp., Armonk, NY, USA). Normality and homogeneity of variances (Levene’s test) of the data was checked using SPSS version 25.0. The survival of DWV-injected honeybees was tested using the Kaplan–Meier survival statistics with log-rank. DWV levels were compared between the normal and crippled wing groups using the Mann–Whitney *U* test. Pearson’s correlation was assessed between the concentrations of DWV injection and number of crippled wings. Moreover, DWV levels in newly emerged adult bees were analyzed using the correlation between concentrations of DWV injection and wing characteristics by Pearson’s correlation. Antimicrobial peptide and apoptosis transcripts in newly emerged adult bees were analyzed by the Kruskal-Wallis test and then post hoc comparison among control, PBS, and wing characteristic in each injected treatment with Bonferroni correction to adjust the probability. In pupae bees, gene transcripts and DWV level were analyzed by the Kruskal-Wallis test, and then post hoc comparison among control and injected treatments with Bonferroni correction to adjust the probability. All tests were considered significant at *p* < 0.05. unless otherwise stated.

## 3. Results

### 3.1. DWV Lysate Type Strain

The DWV genotype in the lysate was the DWV-A genotype with 89.44% homology to the DWV accession number MH069503.1 in the National Center for Biotechnology Information (NCBI) database. DWV-A lysate was used to inject white-eyed honeybee pupae in this study.

### 3.2. Survival of Honeybees from White-Eyed Pupae to Newly Emerged Adult Bees

**Figure 1:**
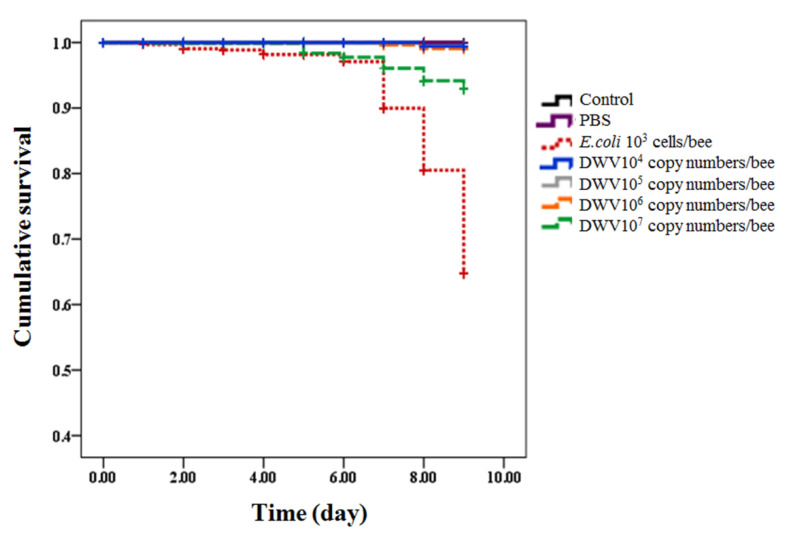
Kaplan–Meier survival curves of honeybee pupae after injecting with *Escherichia coli* (10^3^ cells per bee), initial concentrations of DWV (10^4^, 10^5^, 10^6^, and 10^7^ copy numbers per bee), PBS, and control (un-injected). The survival rates were monitored over a period of 9 days from the pupae stage until newly emerged adult bees.

The survival rate of newly emerged adult bees was 100% in the control group. The survival rates differed amongst treatment groups—100% for PBS; 51% for *E. coli*; and 99%, 99%, 98%, and 81% for DWV at 10^4^, 10^5^, 10^6^, and 10^7^ copy numbers per bee, respectively. The cumulative survival curve of newly emerged adult bees was significantly different within treated groups and control after 9 days post-injection (Kaplan–Meier log-rank test, X^2^ = 287.867, *p*-value < 0.0001). No significant difference in cumulative survival rate was shown amongst the control and treated groups that were injected by PBS and lower three concentrations of DWV (10^4^, 10^5^, and 10^6^ copy numbers per bee) (Table S3). *E. coli*-injected honeybee pupae showed the lowest cumulative survival rate when compared to other groups (log-rank test, all *p*-value < 0.0001) (Figure 1). Honeybee pupae injected with the highest dose (10^7^ copy numbers per bee) showed significantly lower cumulative survival rate than bees in the control group (uninfected), PBS-injected bees, and bees injected with other concentrations of DWV (10^4^, 10^5^, and 10^6^ copy numbers per bee) (log-rank test, all *p*-value < 0.0001, except for 10^6^ copy numbers per bee, which uses *p*-value = 0.001, Table S3).

### 3.3. Crippled Wings in Newly Emerged Adult Bees

After 9 days post-injection, bees in the control and PBS groups showed no crippled wings in their newly emerged adult stage. Almost half of the honeybees (41%) that were injected with *E. coli* showed characteristics of the crippled wing symptoms. At their emergence, honeybees that were injected with DWV at the concentrations of 10^4^, 10^5^, 10^6^, and 10^7^ copy numbers per bee displayed the crippled wing symptoms at 33%, 40%, 51%, and 63% of total newly emerged adult bees, respectively. The number of crippled wings positively correlated with the initial concentration of DWV injection (*r* = 0.855, *p*-value = 0.007).

### 3.4. Amount of DWV in Newly Emerged Adult Bees

**Figure 2:**
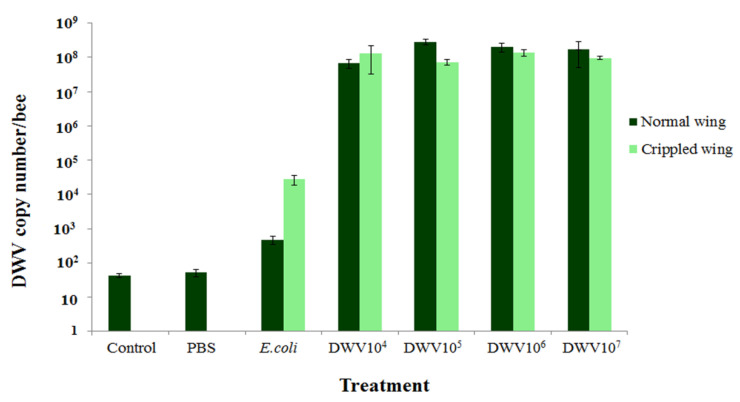
DWV levels in newly emerged adult bees that were injected in their pupae stage with phosphate-buffered saline (PBS), *E. coli* (10^3^ cells per bee), and DWV at concentrations of 10^4^, 10^5^, 10^6^, and 10^7^ copy numbers per bee. Vertical bars represent means ± standard error of the mean (SEM). A comparison between bees that display crippled wing symptoms and those with no symptoms within each treatment group was made using the Mann–Whitney *U* test.

DWV was detected at a very low level (43.7 ± 6.0 and 52.8 ± 13.2 copy numbers per bee) in newly emerged adult bees of the control and PBS groups, respectively, despite the absence of the crippled wing symptoms. The normal newly emerged adult bees that were initially injected with *E. coli* and DWV at concentrations of 10^4^, 10^5^, 10^6^, and 10^7^ copy numbers per bee had DWV levels of 4.8 × 10^2^ ± 1.3 × 10^2^, 6.8 × 10^7^ ± 2.0 × 10^7^, 2.8 × 10^8^ ± 5.0 × 10^7^, 2.0 × 10^8^ ± 5.8 × 10^7^, and 1.7 × 10^8^ ± 1.2 × 10^8^ copy numbers per bee, respectively after 9 days. The cripple winged newly emerged adult bees 9 days post-injection that were initially injected with *E. coli* and DWV at concentrations of 10^4^, 10^5^, 10^6^, and 10^7^ copy numbers per bee had DWV levels of 2.8 × 10^4^ ± 9.0 × 10^3^, 1.3 × 10^8^ ± 9.6 × 10^7^, 7.4 × 10^7^ ± 1.4 × 10^7^, 1.4 × 10^8^ ± 2.7 × 10^7^, and 9.6 × 10^7^ ± 9.6 × 10^6^ copy numbers per bee, respectively. No significant difference was found in the levels of DWV between bees that had crippled wings and those with no symptoms (Mann–Whitney *U*, comparison between normal and crippled wing of *E. coli,* DWV concentration of 10^4^, 10^5^, 10^6^, and 10^7^ copy numbers per bee with *p*-values = 0.05, 0.827, 0.05, 0.268, and 0.827, respectively (Figure 2)). There was also no correlation between DWV levels in newly emerged adult bees and concentrations of DWV injection *(r* = 0.093, *p*-value = 0.667) amongst DWV-injected groups. Moreover, DWV levels were not correlated with wing characteristics in the treated groups (*r* = −0.320, *p*-value = 0.127).

### 3.5. Antimicrobial Peptides and Apoptosis-Related Genes in Newly Emerged Adult Bees

**Figure 3:**
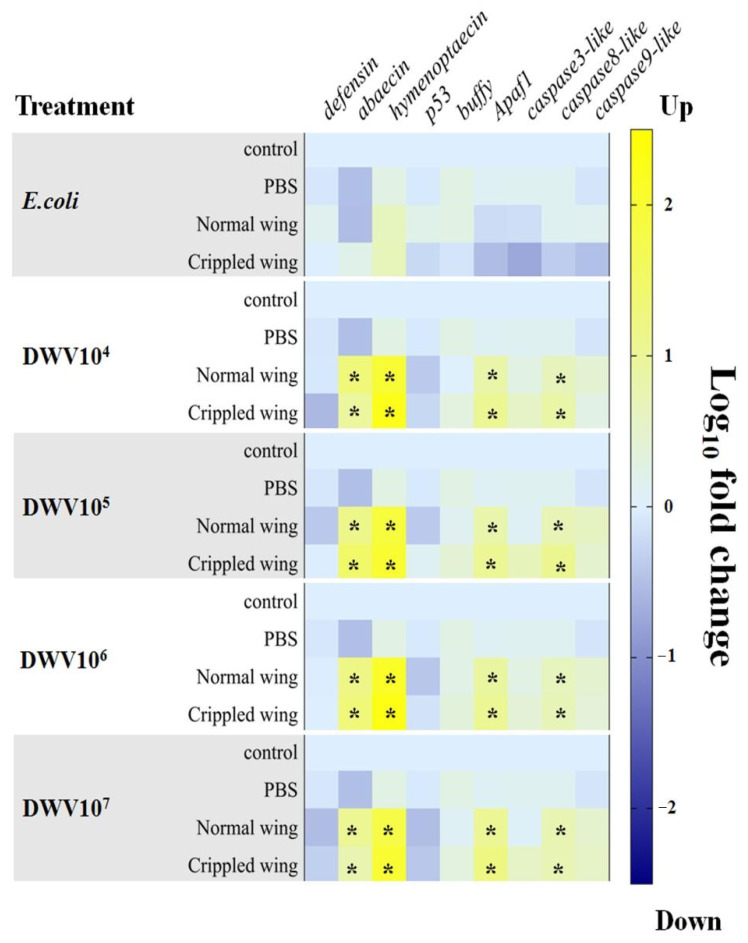
A heatmap of gene expression in newly emerged adult bees after injection with DWV at initial concentrations of 10^4^, 10^5^, 10^6^, and 10^7^ copy numbers per bee, PBS, and *E. coli* (10^3^ cells per bee). Relative fold changes were obtained using the ∆∆Ct method. Ribosomal protein subunit 5 (*RPS5*) and β-actin were used as reference genes. Gene expression in the control, PBS, normal, and crippled wing groups were compared in each treatment. A black asterisk (*) indicates a significant difference when compared with the control and PBS groups (*p* < 0.05; Kruskal-Wallis with Bonferroni correction).

AMP and apoptosis-related gene expression were determined in newly emerged adult bees by injecting the white-eyed pupae with *E. coli* (10^3^ cells per bee) and DWV (10^4^,10^5^, 10^6^, and 10^7^ copy numbers per bee). All genes that were measured in the control and PBS groups were not statistically significantly different amongst groups. The genes *defensin, p53, buffy, caspase3-like,* and *caspase9-like* also were not statistically significantly different between the injected treatment groups and the control and PBS groups. Notably, four genes (*abaecin*, *hymenoptaecin*, *apaf1*, and *caspase8-like*) were statistically significantly upregulated in newly emerged adult bees at all concentrations of injected DWV (Kruskal-Wallis test on gene expression in 10^4^, 10^5^, 10^6^, and 10^7^ copy numbers per bee of DWV-injected bees, *p*-values of *abaecin* = 0.023, 0.023, 0.030, and 0.029, respectively; *p*-values of *hymenoptaecin* = 0.038, 0.036, 0.029, and 0.038, respectively; *p*-values *of apaf1* = 0.033, 0.030, 0.026, and 0.026, respectively; and *p*-values of *caspase8-like* = 0.033, 0.036, 0.036, and 0.038, respectively, in Figure 3). Moreover, the differences in gene expression between normal and crippled wings at all concentrations of DWV were not statistically significant (Kruskal-Wallis with Bonferroni correction *p*-value > 0.05, Table S4). Furthermore, these four genes were not differentially expressed in the group that was injected with *E. coli*.

### 3.6. DWV Levels in Honeybee Pupae Post-Injection

**Figure 4:**
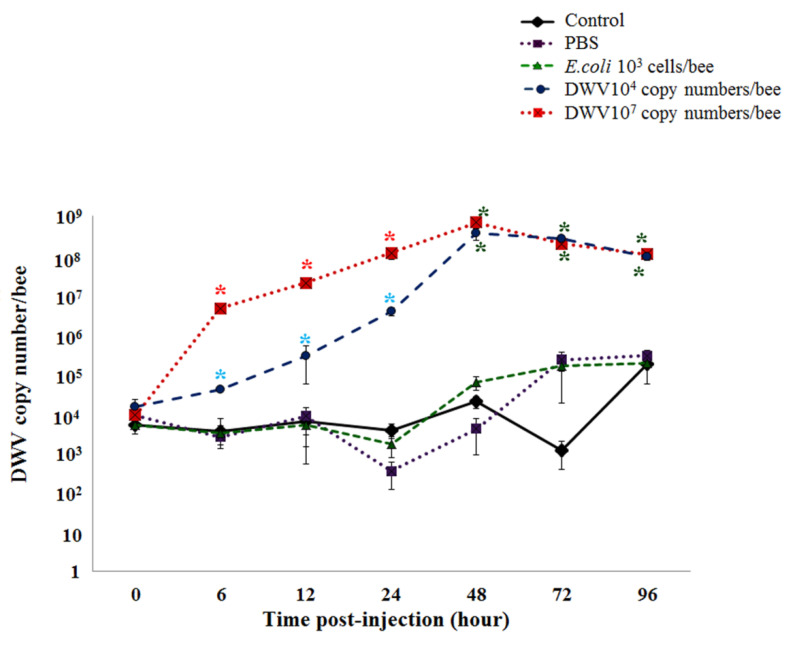
DWV replication kinetics in white-eyed honeybee pupae following injection with DWV at initial concentration of 10^4^ and 10^7^ copy numbers per bee, PBS, and *E. coli* (10^3^ cells per bee). Lines represent the mean ± SD DWV copy number per bee. Significant differences among the injected treatment groups and the control and PBS groups are shown by a black asterisk (*). Significant differences between the injected treatment groups and the PBS group are shown by a blue asterisk (*). A red asterisk (*) shows significant differences among the groups and control, PBS, and *E. coli* groups. (*p*-value < 0.05, Kruskal-Wallis with Bonferroni correction).

White-eyed honeybee pupae were injected with one of the following four treatments: PBS, *E. coli* (10^3^ cells per bee), and DWV (10^4^ or 10^7^ copy numbers per bee) in addition to a control group that was not injected. DWV levels were measured between 0 and 96 h post-infection and showed no statistically significant differences among all treatments at the time of injection (0 h). In addition, the control, PBS, and *E. coli* groups had no statistically significant differences in DWV levels between 6 and 96 h post-injection. At 6, 12, and 24 h, the DWV levels of 10^7^ copy numbers per bee DWV-injected bees increased significantly when compared with the PBS, *E. coli*-injected, and control groups (Kruskal-Wallis with Bonferroni correction *p*-value < 0.05, Table S5). Both groups of pupae that were injected with different concentrations of DWV reached a peak in the DWV copy number per bee 48 h after injection. Between 48 and 96 h, a steady-state level of DWV was observed that was statistically significantly higher than the control and PBS groups (Kruskal-Wallis with Bonferroni correction *p*-value < 0.05, Table S5 and Figure 4).

### 3.7. Antimicrobial Peptides and Apoptosis-Related Genes in Honeybee Pupae

**Figure 5:**
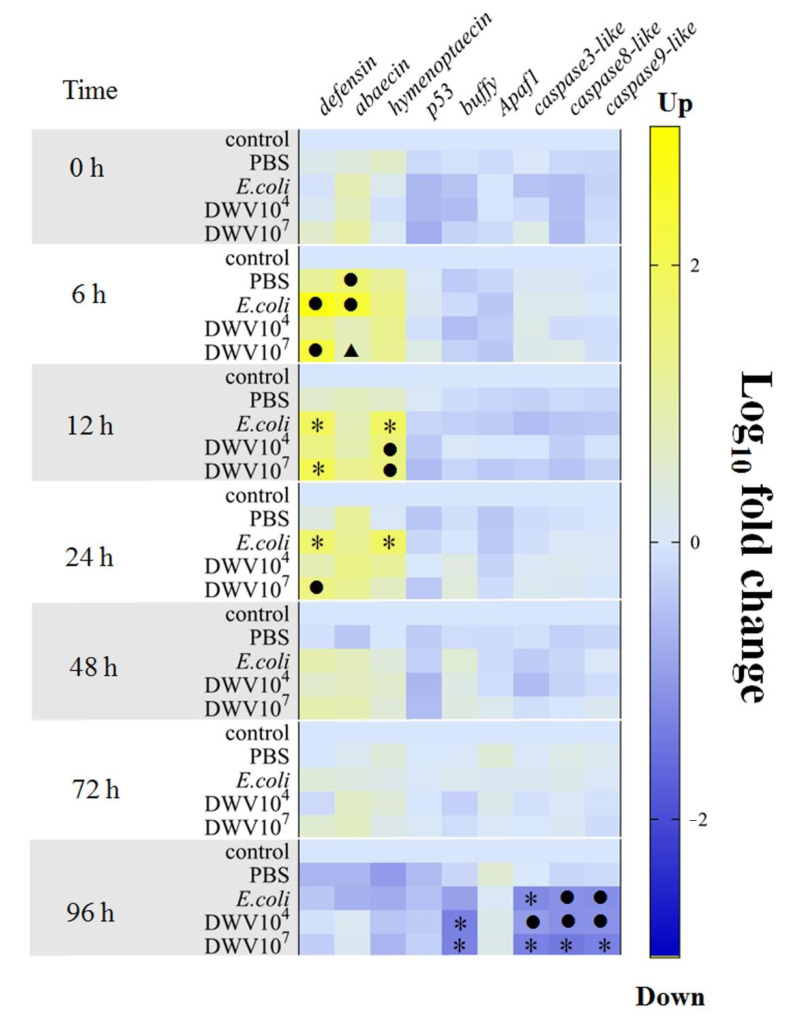
A heatmap of gene expression levels in honeybee pupae over 96 h post-injection with DWV (initial concentrations of 10^4^ or 10^7^ copy numbers per bee), PBS, or *E. coli* (10^3^ cells per bee). Relative fold changes were obtained using the ∆∆Ct method. *RPS5* and β-actin were used as reference genes. The injected treatments were compared with one another in addition to the control group. A black circle (●) indicates a significant difference between the injected treatment and the control. A black triangle (▲) indicates a significant difference between the injected treatment and the *E. coli* group. A black asterisk (*) indicates a significant difference between the injected treatment and the control and PBS groups (*p <* 0.05; Kruskal-Wallis and Bonferroni correction).

The expression of genes encoding AMPs (*defensin*, *abaecin*, and *hymenoptaecin*) was not significantly different at the initial time of injection; however, these genes were upregulated between 6 and 24 h post-injection in the *E. coli* and DWV groups. The levels of *defensin* mRNA were significantly higher within treated groups and control at 6, 12, and 24 h post-injection (Kruskal-Wallis, *p*-value = 0.026, 0.019, and 0.028, respectively). At 6 h, *defensin* was upregulated in *E. coli* group and bees that were injected with 10^7^ DWV copy numbers per bee compared with the controls (Kruskal-Wallis with Bonferroni correction, *p*-value = 0.001 and 0.028, respectively). At 12 and 24 h post-infection, honeybee pupae that were injected with *E. coli* and 10^7^ copy numbers per bee DWV had upregulated *defensin* expression when compared with the control and PBS groups, except 10^7^ copy numbers per bee DWV had significantly higher *defensin* expression than the control alone at 24 h (Kruskal-Wallis with Bonferroni correction, *p*-value < 0.05, Table S6). However, no significant differences in the expression of *defensin* were found among the *E. coli* and DWV groups. Expression of the *abaecin* gene was only significantly higher at 6 h post-infection (Kruskal-Wallis, *p*-value = 0.023). The expression of *abaecin* was significantly upregulated in PBS and *E. coli*-injected pupae when compared with the control group (Kruskal-Wallis with Bonferroni correction, *p*-value = 0.045 and 0.001, respectively). Only the *E. coli*-injected pupae showed higher expression of *abaecin* when compared with the 10^7^ copy numbers per bee DWV group (Kruskal-Wallis with Bonferroni correction, *p*-value = 0.045). The levels of *hymenoptaecin* transcripts were significantly higher in honeybee pupae that were injected with *E. coli* and both concentrations of DWV at 12 h post-injection when compared with the control group (Kruskal-Wallis with Bonferroni correction, *p*-value = 0.008, 0.028, and 0.022, respectively). At 24 h, *hymenoptaecin* was upregulated in the *E. coli* group when compared with the control and PBS groups (Kruskal-Wallis with Bonferroni correction, *p*-value = 0.005 and 0.008, respectively). The expression of *hymenoptaecin* was not significantly different amongst the *E. coli* and two DWV groups (Figure 5).

Changes in the expression of apoptosis-related genes were evaluated by measuring *p53*, *buffy*, *apaf1, caspase3-like*, *caspase8-like*, and *caspase9-like*. The expression of *p53* and *apaf1* did not change significantly across all of the treatment and control groups at all time points. However, four genes (*buffy*, *caspase3-like*, *caspase8-like*, and *caspase9-like*) were downregulated at 96 h post-injection (Kruskal-Wallis, *buffy* gene *p*-value = 0.014, *caspase3-like* gene *p*-value = 0.031, *caspase8-like* gene, *p*-value = 0.032, and *caspase9-like* gene *p*-value = 0.024). The level of *buffy* mRNA was significantly suppressed in DWV-injected pupae (10^4^ and 10^7^ copy numbers per bee) compared with the control (Kruskal-Wallis with Bonferroni correction, *p*-value = 0.003 and 0.014, respectively) and PBS groups (Kruskal-Wallis with Bonferroni correction, *p*-value = 0.044 and 0.014, respectively). The expression of all three *caspase-like* genes (*caspase3-like*, *caspase8-like*, and *caspase9-like*) was significantly downregulated in both DWV-injected pupae groups compared with the control and between the 10^7^ DWV copy numbers per bee and PBS groups (Kruskal-Wallis with Bonferroni correction, *p*-value < 0.05, Table S6). The mRNA levels of the three *caspase-like* genes were not significantly different among the *E. coli* group and both DWV groups at 96 h post-injection (Figure 5).

## 4. Discussion

Our results indicated a low mortality rate during the development of pupae to adult bees following infection. This is in agreement with the findings of Möckel et al. (2011) [38] and Tehel et al. (2019) [39]. In addition, Chen and Siede (2007) reported that pupae were rarely killed by DWV [40]. Viruses largely depend on host cells for replication and transmission [41]; therefore, DWV transmission requires the survival of honeybee pupae. However, adult honeybees that were injected with DWV at a high concentration during their pupal stage showed lower survival rates than pupae that were injected with the lower concentration of DWV.

The present study showed that newly emerged adult bees had the highest proportion of crippled wings when injected with a high concentration (10^7^ copy numbers per bee) during their pupal stages, followed by bees injected with 10^6^, 10^5^, and 10^4^ DWV copy numbers per bee. This result is in accordance with the study by Khongphinitbunjong et al. in 2016 that showed the proportion of deformed bees numerically increased with increasing DWV concentrations that were administered by feeding two-day-old larvae with DWV lysate. This finding could be attributed to the increased frequency of infection and multiplication of DWV in localized tissues during development following infection with high concentrations of DWV [33]. Furthermore, a high frequency of crippled wings was also noted in bees that were injected with *E. coli*. We suggested that a deterioration in honeybee health and covert DWV replication after *E. coli* injection could account for this observation [42].

DWV was detected in three experimental groups: the control, PBS, and *E. coli*-injected bee groups; however, it was found at a low level. In these groups, low levels of DWV might have occurred through transmission from mother to offspring (vertical transmission) [43] or contamination in the semen of drones [44]. In addition, the queen can naturally mate with many different drones [45]. Therefore, DWV is commonly found in honeybee broods. However, in this study, newly emerged adult bees that were injected with DWV during the pupal stages had significantly higher DWV levels (6.8 × 10^7^ to 2.8 × 10^8^ copy numbers per bee) than the control, PBS, and *E. coli*-injected groups. No statistically significant differences in DWV levels were found between normal and crippled wing newly emerged adult bees and among groups that had different concentrations of DWV injected during the pupal stages. Therefore, it is possible that the observed wing deformity was not related to DWV levels. Dubois et al. also reported similar viral loads in honeybees with deformed- and normal-winged pupae [29]. Our result also revealed that there was no correlation between deformed wings in DWV-injected bees and the response of immune- and apoptosis-related genes examined in this study. Disease symptoms might rely on other factors such as the time of infection, viral strains, routes of transmission, and host vulnerability [46,47]. Moreover, disease symptoms can occur due to environmental factors such as subnormal temperatures during pupal development [48]. Interestingly, viral susceptibility and disease progression have been associated with host genetic variation of individual differences. For example, *Drosophila melanogaster* was investigated for the presence of host resistance genes. In particular, it was reported that the ref(2)P polymorphism in *D. melanogaster* was important to viral susceptibility and linked to the innate immunity of Toll signaling and autophagy pathways [49,50]. Individual honeybees may also possess polymorphic genes that might be linked to resistance or susceptibility to DWV infection and related to disease symptoms. In this experiment, we only measured the quantity of DWV and not post-translational outcomes such as capsid assembly, nucleic acid packaging, and virus particle maturation. Therefore, additional factors contributing to wing deformity should be further investigated as these processes may contribute to disease appearance and host defense.

This study examined the effect of different initial concentrations of DWV injections at the pupal stages. The data revealed that 10^4^ and 10^7^ DWV copy numbers per bee replicated at the highest level within 48 h. The viral levels showed no significant difference between both concentrations after 48 h and reached a steady state after 96 h. This indicated that DWV exists as a persistent infection [14] and likely control individual host defense against the pathogen.

This is the first study to report a time-series gene expression analysis of DWV during infection in the pupal stage of honeybees. *E. coli* was used as a positive control because it is known to activate the humoral immune response and produces a well-described antimicrobial immune response in honeybees [27,28,42]. The expression of AMP genes was upregulated within 24 h of DWV and *E. coli* infection of honeybee pupae, which reflected the hosts’ response to microbial infections. Many reports have shown that AMP genes in honeybees were upregulated after invasion of pathogens including *Nosema* [51], *Paenibacillus larvae* [52], fungi [53], viruses, and bee mites [54,55]. AMPs are non-specific innate immune responses to a broad-spectrum of pathogens [56]. Therefore, AMPs were rapidly upregulated when honeybees were infected with DWV and *E. coli*. *E. coli* is a non-pathogenic bacterium that was killed by the hosts’ immune response. A study of the immune responses of five standard *D. melanogaster* strains (Oregon R, w1118, Canton-S, Cinnabar Brown, and Yellow White) to *E. coli* infection reported no detectable levels of *E. coli* cells in all strains 24 h after infection [57]. In addition, AMPs were significantly higher between 6 and 12 h post-infection but had reduced by 48 h [57]. Therefore, this mechanism could account for the lack of AMP expression in *E. coli*-infected bees between 48 h and 9 days post-injection in our study. However, AMPs are known to be more strongly activated by bacterial infection than viral infection. It has been reported that lipopolysaccharides (LPS) in the outer membrane of Gram-negative bacteria were strong stimulators of innate immunity in insects and humans [58]. Hence, *E. coli*-injected honeybees had a stronger immune activation than DWV-injected honeybees (*abaecin* at 6 h). Innate immune responses can be induced by viruses within the host via viral entry, replication, and spreading [59]. RNA-dependent RNA polymerases (RdRPs), which replicate the viral RNA genome in picornaviruses, can activate mammalian innate immunity systems. Transgenic mouse and human cell culture models that express a viral RNA-dependent RNA polymerase (RdRP) showed a high elevation of interferon-stimulated gene (ISG) expression of up to 300-fold and 560-fold, respectively [60]. It is possible that AMPs were induced in this study when the number of DWV copies increased during the early infection of pupae. In our study, we found that the upregulation of transcript levels of immune-related genes peaked within 24 h post-infection while the DWV copy numbers in infected bees peaked after 48 h. The DWV copy numbers persisted at a steady state at 48, 72, and 96 h post-infection. Moreover, the persistence of RNA viruses can avoid elimination by host immune responses. For example, it has been reported that miR-122 binds to the genome of HCV to protect it from 5′–3′ exoribonuclease Xrn1 degradation and inhibits detection by sensors of the innate immune response [61]. Therefore, the AMP response might be not induced in this stage. In newly emerged adult bees, it is possible that DWV genomes in the persistence stage were assembled into virus particles and spread from one cell to neighboring cells. This might be the cause of host immune stimulation in newly emerged bees [59]. However, further studies to investigate the viral life cycle stage and pathogenesis are needed as viral particles were not directly measured in this study.

It is known that viruses can avoid programmed cell death by suppression of caspases, which are the final proteases in apoptosis [22]. In this study, the suppression of *caspase3-like*, *caspase8-like*, and *caspase9-like* in DWV-injected honeybee pupae was investigated at 96 h. We suggested that this virus might block apoptosis pathways despite the downregulation of transcripts encoding Buffy, which is Bcl-2-like pro-survival protein displaying anti-apoptotic and cell cycle inhibitory functions [21,62,63]. Similarly, Erban et al. in 2019 reported that anti-apoptotic Bcl-2 transcripts were downregulated in DWV-infected honeybees and *Varroa*-infested bees with visible DWV symptoms [26]. The *p53* gene has also been demonstrated to downregulate Bcl-2 protein and triggered apoptosis cascades [64,65]. Studies show that different viruses manipulate *p53* in different strategies to proliferate in host cells [66]. Moreover, the covert DWV infection in honeybees that was activated by *E. coli* [42] might have occurred owing to apoptosis-changing events arising from the *E.coli* injection treatment. However, covert DWV in nature is categorized as a low virulence virus [44]. In this study, the late upregulation of *apaf1* and *caspase* (particularly *caspase8-like*) gene expression could have occurred through the activation of apoptosis during DWV persistence in infected bees. It is also possible that DWV genomes in the persistence phase could form virus particles in the late stages of infection and activate cellular apoptosis. Furthermore, DWV is non-enveloped and non-enveloped viruses typically cause cell lysis [67]. Therefore, the virus could activate cell death by releasing viral progeny or virus dissemination. Enteroviruses (EV) are positive-sense RNA viruses in the order Picornavirales [68] that have been well studied in relation to apoptosis. EV can inhibit apoptotic pathways in the early stage of infection but induce apoptosis to release viral progeny in the late stage of infection [69]. Therefore, it is reasonable to suggest that the apoptosis mechanism may be used to destroy the cell and release DWV virus particles in newly emerged adult bees and further induce immune responses within the host [59]. This could result in the upregulation of immune- (*abaecin* and *hymenoptaecin*) and apoptosis-related genes in newly emerged adult bees.

## 5. Conclusions

In this study, DWV caused low mortality in newly emerged adult bees that were injected during the pupal stage. The number of bees with crippled wings in the newly emerged adult bee population was directly dependent on the initial concentration of DWV that was injected during the pupal stage. However, DWV levels and immune- and apoptosis-related gene expression in newly emerged adult bees did not seem to affect the wing deformity of bees. Immune genes were upregulated against DWV infection in pupae and newly emerged adult bees. DWV persistence might avoid individual bee defenses by suppression of apoptosis-related genes such as *caspase3-like, caspase8-like*, and *caspase9-like* to improve the survival of DWV in honeybee pupae. However, an upregulation of apoptosis-related genes in newly emerged adult bees could reflect a stimulation of apoptosis to enhance the release of new DWV particles during the adult stage of bees.

## Figures and Tables

**Table 1:** Primers sequences and characteristics of the RT-qPCR amplification in this study for deformed wing virus (DWV) injection experiment.

Target Gene Amplified	Primer Name	Sequence 5′-3′	References
**Reference gene**			
**Ribosomal protein S5 (RPS5)**	AmRPS5.F	AATTATTTGGTCGCTGGAATTG	[35]
	AmRPS5.R	TAACGTCCAGCAGAATGTGGTA	
**β-Actin**	Actin.F	TTGTATGCCAACACTGTCCTTT	[36]
	Actin.R	TGGCGCGATGATCTTAATTT	
**Immune-related gene (antibacterial peptide)**			
**Abaecin**	Abaecin.F	CAGCATTCGCATACGTACCA	[25]
	Abaecin.R	GACCAGGAAACGTTGGAAAC	
**Defensin**	Defensin.F	TGCGCTGCTAACTGTCTCAG	[25]
	Defensin.R	AATGGCACTTAACCGAAACG	
**Hymenoptaecin**	Hymenopt.F	CTCTTCTGTGCCGTTGCATA	[25]
	Hymenopt.R	GCGTCTCCTGTCATTCCATT	
**Apoptosis-related gene**			
**Apoptotic peptidase activating factor 1-like**	Apaf1.F	ACAGATGATAATTTACAGGTGTGGG	NC_007071.3
	Apaf1.R	TCCGTTCACTCTATCCGTTTGT	
**Bcl-2 family proteins-like**	Buffy.F	TGCCGATGCCTGAAAAGTCT	NC_007072.3
	Buffy.R	TCTGCGATAAGGTTGGCCTG	
**Tumor protein p53-inducible nuclear protein 2-like**	P53.F	TTCAATTGCACAGTTGAGGGC	NW_003382505.1
	P53.R	CGGTACACGGACATACTGGG	
**Cysteine proteases3-like**	Caspase3-like.F	CATGCACAGAAGAAATTCGCCA	XM_394855.6
	Caspase3-like.R	GTTCGTCCCGTTTCGTTGTG	
**Cysteine proteases8-like**	Caspase8-like.F	AAAACAATTGATGCAGTAGGGG	XM_006570913.3
	Caspase8-like.R	TTCTGGAAATTGAAAATCGGAAGA	
**Cysteine proteases9-like**	Caspase9-like.F	TGGCCAAAGCTTGTTGAAAATCA	XM_026444610.1
	Caspase9-like.R	ATGCAAAAGGTCCCCGTGTT	
**Honeybee virus**			
**Deformed wing virus**	DWV.F	CGAAACCAACTTCTGAGGAA	[37]
	DWV.R	GTGTTGATCCCTGAGGCTTA	

## Data Availability

Not applicable.

## Supplementary Materials

The following are available online at https://www.mdpi.com/2075-4450/12/1/82/s1: Table S1. Primer sequences of seven honeybee virus detections. Table S2. Honeybee virus detections (Ct values) in DWV lysate. Table S3. The statistical test of survival in newly emerged adult bees. Table S4. The statistical tests of gene expression in newly emerged adult bees. Table S5. The statistical tests of DWV levels in honeybee pupae. Table S6. The statistical tests of gene expression in honeybee pupae.
