# Supplementary material for: Effects of Deformed Wing Virus Infection on Expressions of Immune- and Apoptosis-Related Genes in Western Honeybees (Apis mellifera)
Source: Insects. 2021 Jan 19;12(1):82. doi: 10.3390/insects12010082 (PMC7832323; doi:10.3390/insects12010082)
Supplement: Supplementary file 1 [file insects-12-00082-s001.pdf]

Table S1. Primer sequences of seven honeybee virus detections

Primer Name	Sequence 5'-3'	Produce size (bp)
BQCV	AGTGGCGGAGATGTATGC	294
	GGAGGTGAAGTGGCTATATC	
KBV	CCATACCTGCTGATAACC	200
	CTGAATAATACTGTGCGTATC	
SBV	GCTCTAACCTCGCATCAAC	335
	TTGGAACTACGCATTCTCTG	
ABPV	TCATACCTGCCGATCAAG	197
	CTGAATAATACTGTGCGTATC	
CBPV	CAACCTGCCTCAACACAG	296
	AATCTGGCAAGGTTGACTGG	
IAPV	CCATGCCTGGCGATTCAC	203
	CTGAATAATACTGTGCGTATC	

Table S2. Honeybee virus detections (Ct values) in DWV lysate

Virus detection	Ct value ± SD
Deformed wing virus	13.51±0.61
Acute bee paralysis virus	40.00±0.00
Black queen cell virus	40.00±0.00
Chronic bee paralysis virus	40.00±0.00
Israeli acute paralysis virus	40.00±0.00
Kashmir bee virus	40.00±0.00
Sacbrood virus	40.00±0.00

Table S3. The statistical test of survival in newly emerged adult bees

**Treatments**	**Log-rank test:X^2 and *p-value***						
	control	PBS	*E.coli* 10^3	DWV 10^4	DWV 10^5	DWV 10^6	DWV 10^7
control		-	74.217	1.006	1.006	2.028	17.953
PBS	-		74.217	1.006	1.006	2.028	17.953
E.coli 10^3	**<0.0001***	**<0.0001***		70.334	136.558	66.727	24.658
DWV 10^4	0.316	0.316	**<0.0001***		0.000	0.517	16.613
DWV 10^5	0.316	0.316	**<0.0001***	1.000		0.334	16.477
DWV 10^6	0.154	0.154	**<0.0001***	0.472	0.558		10.864
DWV 10^7	**<0.0001***	**<0.0001***	**<0.0001***	**<0.0001***	**<0.0001***	**0.001***	

- = not a statistical different test

* = the statistically significant difference at *p-value* < 0.05

Table S4. The statistical tests of gene expression in newly emerged adult bees

Defensin			***Abaecin***						
**Treatment**	Kruskal-Wallis		**Treatment**	Kruskal-Wallis		Pairwise Comparisons of treatment (Significance values have been adjusted by the Bonferroni correction for multiple tests)			
	H	***p-value***		H	***p-value***	**Treatment**		H	***p-value***
E.coli	1.402	0.705	*E.coli*	3.205	0.361	-		-	-
DWV10^4	4.538	0.209	DWV10^4	9.564	**0.023***	Control	PBS	-2.333	0.428
						Control	Normal	6.000	**0.042***
						Control	Cripple	5.667	**0.049***
						PBS	Normal	8.333	**0.005***
						PBS	Cripple	6.000	**0.042***
						Normal	Cripple	-2.333	0.428
DWV10^5	5.359	0.147	DWV10^5	9.564	**0.023***	Control	PBS	-2.333	0.428
						Control	Normal	5.667	**0.049***
						Control	Cripple	6.000	**0.042***
						PBS	Normal	6.000	**0.042***
						PBS	Cripple	8.333	**0.005***
						Normal	Cripple	2.333	0.428
DWV10^6	0.064	0.996	DWV10^6	8.949	**0.030***	Control	PBS	-2.333	0.428
						Control	Normal	6.000	**0.042***
						Control	Cripple	6.000	**0.042***
						PBS	Normal	7.000	**0.017***
						PBS	Cripple	7.333	**0.013***
						Normal	Cripple	0.333	0.910
DWV10^7	3.718	0.294	DWV10^7	9.051	**0.029***	Control	PBS	-2.333	0.428
						Control	Normal	6.000	**0.042***
						Control	Cripple	6.000	**0.042***
						PBS	Normal	7.667	**0.009***
						PBS	Cripple	6.667	**0.024***
						Normal	Cripple	-1.000	0.734

- = not a statistical different test

* = the statistically significant difference at *p-value* < 0.05

Table S4. The statistical tests of gene expression in newly emerged adult bees

Hymenoptaecin							***P53***		
**Treatment**	Kruskal-Wallis		Pairwise Comparisons of treatment (Significance values have been adjusted by the Bonferroni correction for multiple tests)				**Treatment**	Kruskal-Wallis	
	H	***p*-value**	Treatment		H	*p*-value		H	***p*-value**
E.coli	3.821	0.282	-		-	-	*E.coli*	1.570	0.666
DWV10^4	8.436	**0.038***	Control	PBS	1.000	0.734	DWV10^4	7.694	0.053
			Control	Normal	6.333	**0.031***			
			Control	Cripple	6.667	**0.024***			
			PBS	Normal	5.870	**0.048***			
			PBS	Cripple	6.220	**0.041***			
			Normal	Cripple	0.333	0.910			
DWV10^5	8.538	**0.036***	Control	PBS	1.000	0.734	DWV10^5	4.657	0.199
			Control	Normal	6.000	**0.042***			
			Control	Cripple	7.000	**0.017***			
			PBS	Normal	6.000	**0.042***			
			PBS	Cripple	6.000	**0.042***			
			Normal	Cripple	1.000	0.734			
DWV10^6	9.051	**0.029***	Control	PBS	1.000	0.428	DWV10^6	1.467	0.690
			Control	Normal	6.000	**0.042***			
			Control	Cripple	7.667	**0.009***			
			PBS	Normal	6.000	**0.042***			
			PBS	Cripple	6.667	**0.024***			
			Normal	Cripple	2.333	0.734			
DWV10^7	8.436	**0.038***	Control	PBS	1.000	0.734	DWV10^7	5.841	0.120
			Control	Normal	6.333	**0.031***			
			Control	Cripple	6.667	**0.024***			
			PBS	Normal	5.870	**0.048***			
			PBS	Cripple	6.220	**0.041***			
			Normal	Cripple	0.333	0.910			

- = not a statistical different test

* = the statistically significant difference at *p-value* < 0.05

Table S4. The statistical tests of gene expression in newly emerged adult bees

Buffy			***Apaf1***						
**Treatment**	Kruskal-Wallis		**Treatment**	Kruskal-Wallis		Pairwise Comparisons of treatment (Significance values have been adjusted by the Bonferroni correction for multiple tests)			
	H	***p*-value**		H	***p*-value**	**Treatment**		H	***p*-value**
E.coli	3.718	0.070	*E.coli*	5.256	0.154	-			-
DWV10^4	2.949	0.400	DWV10^4	8.744	**0.033***	Control	PBS	1.667	0.571
						Control	Normal	6.333	**0.031***
						Control	Cripple	7.333	**0.013***
						PBS	Normal	5.667	**0.049***
						PBS	Cripple	6.000	**0.042***
						Normal	Cripple	1.000	0.734
DWV10^5	4.231	0.238	DWV10^5	8.949	**0.030***	Control	PBS	1.667	0.571
						Control	Normal	6.000	**0.042***
						Control	Cripple	7.667	**0.009***
						PBS	Normal	7.210	**0.014***
						PBS	Cripple	6.000	**0.042***
						Normal	Cripple	1.667	0.571
DWV10^6	5.359	0.147	DWV10^6	9.256	**0.026***	Control	PBS	1.667	0.571
						Control	Normal	5.667	**0.049***
						Control	Cripple	8.000	**0.007***
						PBS	Normal	5.667	**0.049***
						PBS	Cripple	6.333	**0.031***
						Normal	Cripple	2.333	0.428
DWV10^7	2.590	0.459	DWV10^7	9.256	**0.026***	Control	PBS	1.667	0.571
						Control	Normal	5.667	**0.049***
						Control	Cripple	8.000	**0.007***
						PBS	Normal	5.667	**0.049***
						PBS	Cripple	6.333	**0.031***
						Normal	Cripple	2.333	0.428

- = not a statistical different test

* = the statistically significant difference at *p-value* < 0.05

Table S4. The statistical tests of gene expression in newly emerged adult bees

Caspase3-like			***Caspase8-like***						
**Treatment**	Kruskal-Wallis		**Treatment**	Kruskal-Wallis		Pairwise Comparisons of treatment (Significance values have been adjusted by the Bonferroni correction for multiple tests)			
	H	***p*-value**		H	***p*-value**	Treatment		H	*p*-value
E.coli	7.308	0.063	*E.coli*	4.644	0.200	-		-	-
DWV10^4	5.154	0.161	DWV10^4	8.744	**0.033***	Control	PBS	-1.000	0.734
						Control	Normal	7.550	**0.011***
						Control	Cripple	6.333	**0.031***
						PBS	Normal	5.667	**0.049***
						PBS	Cripple	7.333	**0.013***
						Normal	Cripple	1.667	0.571
DWV10^5	5.256	0.154	DWV10^5	8.538	**0.036***	Control	PBS	-1.000	0.734
						Control	Normal	6.000	**0.042***
						Control	Cripple	6.000	**0.042***
						PBS	Normal	6.000	**0.042***
						PBS	Cripple	7.000	**0.017***
						Normal	Cripple	1.000	0.734
DWV10^6	3.359	0.340	DWV10^6	8.538	**0.036***	Control	PBS	-1.000	0.734
						Control	Normal	6.000	**0.042***
						Control	Cripple	6.000	**0.042***
						PBS	Normal	6.000	**0.042***
						PBS	Cripple	7.000	**0.017***
						Normal	Cripple	1.000	0.734
DWV10^7	2.590	0.459	DWV10^7	8.436	**0.038***	Control	PBS	-1.000	0.734
						Control	Normal	6.220	**0.041***
						Control	Cripple	5.870	**0.048***
						PBS	Normal	6.667	**0.024***
						PBS	Cripple	6.333	**0.031***
						Normal	Cripple	-0.333	0.910

- = not a statistical different test

* = the statistically significant difference at *p-value* < 0.05

Table S5. The statistical tests of DWV levels in honeybee pupae

**Time**(h)	Kruskal-Wallis		Pairwise Comparisons of treatment (Significance values have been adjusted by the Bonferroni correction for multiple tests)			
	H	***p*-value**	**Treatment**		H	***p*-value**
0	5.600	0.231	-		-	
6	11.500	**0.021***	Control	PBS	3.000	0.411
			Control	*E.coli*	1.000	0.784
			Control	$DWV10^4$	-4.667	0.201
			Control	$DWV10^7$	-7.667	**0.036***
			PBS	*E.coli*	-2.000	0.584
			PBS	$DWV10^4$	-7.667	**0.036***
			PBS	$DWV10^7$	-10.667	**0.003***
			E.coli	$DWV10^4$	-5.667	0.121
			E.coli	$DWV10^7$	-8.667	**0.018***
			$DWV10^4$	$DWV10^7$	-3.000	0.411
12	11.314	**0.023***	Control	PBS	0.667	0.855
			Control	*E.coli*	-1.667	0.647
			Control	$DWV10^4$	-6.333	0.082
			Control	$DWV10^7$	-9.333	**0.010***
			PBS	*E.coli*	-2.333	0.521
			PBS	$DWV10^4$	-7.000	**0.048***
			PBS	$DWV10^7$	-10.000	**0.006***
			E.coli	$DWV10^4$	-4.667	0.200
			E.coli	$DWV10^7$	-7.667	**0.035***
			$DWV10^4$	$DWV10^7$	-3.000	0.410
24	11.500	**0.021***	Control	PBS	3.000	0.411
			Control	*E.coli*	2.000	0.584
			Control	$DWV10^4$	-4.333	0.235
			Control	$DWV10^7$	-7.333	**0.045***
			PBS	*E.coli*	-1.000	0.784
			PBS	$DWV10^4$	-7.333	**0.045***
			PBS	$DWV10^7$	-10.333	**0.005***
			E.coli	$DWV10^4$	-6.333	0.083
			E.coli	$DWV10^7$	-9.333	**0.010***
			$DWV10^4$	$DWV10^7$	-3.000	0.411

- = not a statistical different test

* = the statistically significant difference at *p-value* < 0.05

Table S5. The statistical tests of DWV levels in honeybee pupae

**Time** (h)	Kruskal-Wallis		Pairwise Comparisons of treatment (Significance values have been adjusted by the Bonferroni correction for multiple tests)			
	H	***p*-value**	Treatment		H	*p-value*
48	12.656	**0.013***	Control	PBS	1.000	0.784
			Control	*E.coli*	-4.000	0.273
			Control	DWV10^4	-7.333	**0.044***
			Control	DWV10^7	-9.667	**0.008***
			PBS	*E.coli*	-5.000	0.171
			PBS	DWV10^4	-8.333	**0.022***
			PBS	DWV10^7	-10.667	**0.003***
			E.coli	DWV10^4	-3.333	0.361
			E.coli	DWV10^7	-5.667	0.120
			DWV10^4	DWV10^7	-2.333	0.522
72	12.564	**0.014***	Control	PBS	-0.333	0.927
			Control	*E.coli*	-4.667	0.201
			Control	DWV10^4	-10.333	**0.005***
			Control	DWV10^7	-8.000	**0.028***
			PBS	*E.coli*	-4.333	0.235
			PBS	DWV10^4	-10.000	**0.006***
			PBS	DWV10^7	-7.667	**0.036***
			E.coli	DWV10^4	-5.667	0.121
			E.coli	DWV10^7	-3.333	0.361
			DWV10^4	DWV10^7	2.333	0.523
96	12.030	**0.017***	Control	PBS	-3.167	0.385
			Control	*E.coli*	-4.833	0.185
			Control	DWV10^4	-9.667	**0.008***
			Control	DWV10^7	-10.667	**0.003***
			PBS	*E.coli*	-1.667	0.648
			PBS	DWV10^4	-7.333	**0.045***
			PBS	DWV10^7	-7.500	**0.040***
			E.coli	DWV10^4	-4.833	0.185
			E.coli	DWV10^7	-5.833	0.110
			DWV10^4	DWV10^7	-1.000	0.784

- = not a statistical different test

* = the statistically significant difference at *p-value* < 0.05

Table S6. The statistical tests of gene expression in honeybee pupae

Defensin						
**Time** (h)	Kruskal-Wallis		Pairwise Comparisons of treatment (Significance values have been adjusted by the Bonferroni correction for multiple tests)			
	H	***p*-value**	**Treatment**		H	***p*-value**
0	2.533	0.639	-		-	-
6	11.033	**0.026***	Control	PBS	5.333	0.144
			Control	*E.coli*	11.667	**0.001***
			Control	DWV10^4	5.000	0.171
			Control	DWV10^7	8.000	**0.028***
			PBS	*E.coli*	6.333	0.083
			PBS	DWV10^4	-0.333	0.927
			PBS	DWV10^7	2.667	0.465
			E.coli	DWV10^4	-6.667	0.068
			E.coli	DWV10^7	-3.667	0.315
			DWV10^4	DWV10^7	3.000	0.411
12	11.767	**0.019***	Control	PBS	2.333	0.523
			Control	*E.coli*	9.667	**0.008***
			Control	DWV10^4	6.333	0.083
			Control	DWV10^7	10.000	**0.006***
			PBS	*E.coli*	7.333	**0.045***
			PBS	DWV10^4	4.000	0.273
			PBS	DWV10^7	7.667	**0.036***
			E.coli	DWV10^4	-3.333	0.361
			E.coli	DWV10^7	0.333	0.927
			DWV10^4	DWV10^7	3.667	0.315

Abaecin						
**Time** (h)	Kruskal-Wallis		Pairwise Comparisons of treatment (Significance values have been adjusted by the Bonferroni correction for multiple tests)			
	H	*p*-value	**Treatment**		H	***p*-value**
0	2.933	0.569	-		-	-
6	11.333	**0.023***	Control	PBS	7.333	**0.045***
			Control	*E.coli*	12.000	**0.001***
			Control	DWV10^4	6.000	0.100
			Control	DWV10^7	4.667	0.201
			PBS	*E.coli*	4.667	0.201
			PBS	DWV10^4	-1.333	0.715
			PBS	DWV10^7	-2.667	0.465
			E.coli	DWV10^4	-6.000	0.100
			E.coli	DWV10^7	-7.333	**0.045***
			DWV10^4	DWV10^7	-1.333	0.715
12	3.567	0.468	-		-	

- = not a statistical different test

* = the statistically significant difference at *p-value* < 0.05

Table S6. The statistical tests of gene expression in honeybee pupae

Defensin							***Abaecin***					
**Time** (h)	Kruskal-Wallis		Pairwise Comparisons of treatment (Significance values have been adjusted by the Bonferroni correction for multiple tests)				**Time** (h)	Kruskal-Wallis		Pairwise Comparisons of treatment (Significance values have been adjusted by the Bonferroni correction for multiple tests)		
	H	***p*-value**	**Treatment**		H	***p*-value**		*H*	*p*-value	**Treatment**	H	***p*-value**
24	10.900	**0.028***	Control	PBS	2.333	0.523	24	7.567	0.109		-	-
			Control	*E.coli*	10.000	**0.006***						
			Control	DWV10^4	5.333	0.144						
			Control	DWV10^7	9.000	**0.014***						
			PBS	*E.coli*	7.667	**0.036***						
			PBS	DWV10^4	3.000	0.411						
			PBS	DWV10^7	6.667	0.068						
			E.coli	DWV10^4	-4.667	0.201						
			E.coli	DWV10^7	-1.000	0.784						
			DWV10^4	DWV10^7	3.667	0.315						
48	6.233	0.182	-		-	-	48	5.033	0.284	-	-	-
72	9.200	0.056	-		-	-	72	5.184	0.269	-	-	-
96	5.368	0.252	-		-	-	96	5.200	0.267	-	-	-

- = not a statistical different test

* = the statistically significant difference at *p-value* < 0.05

Table S6. The statistical tests of gene expression in honeybee pupae

Hymenoptaecin							***P53***		
**Time** (h)	Kruskal-Wallis		Pairwise Comparisons of treatment (Significance values have been adjusted by the Bonferroni correction for multiple tests)				**Time** (h)	Kruskal-Wallis	
	H	***p*-value**	**Treatment**		H	*p*-value		*H*	***p*-value**
0	7.500	0.112	-		-	-	0	8.967	0.062
6	7.033	0.134	-		**-**	-	6	0.933	0.920
12	10.767	**0.029***	Control	PBS	2.333	0.523	12	8.033	0.090
			Control	*E.coli*	9.667	**0.008***			
			Control	DWV10^4	8.000	**0.028***			
			Control	DWV10^7	8.333	**0.022***			
			PBS	*E.coli*	7.333	**0.045***			
			PBS	DWV10^4	5.667	0.121			
			PBS	DWV10^7	6.000	0.100			
			E.coli	DWV10^4	-1.667	0.648			
			E.coli	DWV10^7	-1.333	0.715			
			DWV10^4	DWV10^7	0.333	0.927			
24	11.067	**0.026***	Control	PBS	0.667	0.855	24	8.433	0.077
			Control	*E.coli*	10.333	**0.005***			
			Control	DWV10^4	4.000	0.273			
			Control	DWV10^7	6.667	0.068			
			PBS	*E.coli*	9.667	**0.008***			
			PBS	DWV10^4	3.333	0.361			
			PBS	DWV10^7	6.000	0.100			
			E.coli	DWV10^4	-6.333	0.083			
			E.coli	DWV10^7	-3.667	0.315			
			DWV10^4	DWV10^7	2.667	0.465			

- = not a statistical different test

* = the statistically significant difference at *p-value* < 0.05

Table S6. The statistical tests of gene expression in honeybee pupae

Hymenoptaecin						***P53***		
**Time** (h)	Kruskal-Wallis		Pairwise Comparisons of treatment (Significance values have been adjusted by the Bonferroni correction for multiple tests)			**Time** (h)	Kruskal-Wallis	
	H	***p*-value**	**Treatment**	H	*p*-value		H	***p*-value**
48	4.633	0.327	-	-	-	48	6.433	0.169
72	6.779	0.148	-	-	-	72	0.192	0.996
96	9.317	0.054	-	-	-	96	7.967	0.093

- = not a statistical different test

* = the statistically significant difference at *p-value* < 0.05

Table S6. The statistical tests of gene expression in honeybee pupae

Buffy							***Apaf1***		
**Time** (h)	Kruskal-Wallis		Pairwise Comparisons of treatment (Significance values have been adjusted by the Bonferroni correction for multiple tests)				**Time** (h)	Kruskal-Wallis	
	H	*p*-value	Treatment		H	*p*-value		H	*p*-value
0	9.033	0.060	-		-	-	0	4.300	0.367
6	4.784	0.310	-		-	-	6	2.433	0.657
12	3.033	0.552	-		-	-	12	4.500	0.343
24	6.654	0.155	-		-	-	24	7.767	0.101
48	5.167	0.271	-		-	-	48	2.167	0.705
72	6.600	0.159	-		-	-	72	2.067	0.723
96	12.567	**0.014***	Control	PBS	-1.667	0.648	96	3.967	0.411
			Control	*E.coli*	-5.333	0.144			
			Control	DWV10^4	-10.667	**0.003***			
			Control	DWV10^7	-9.000	**0.014***			
			PBS	*E.coli*	-3.667	0.315			
			PBS	DWV10^4	-7.333	**0.044***			
			PBS	DWV10^7	-9.000	**0.014***			
			E.coli	DWV10^4	-5.333	0.144			
			E.coli	DWV10^7	-3.667	0.315			
			DWV10^4	DWV10^7	1.667	0.648			

- = not a statistical different test

* = the statistically significant difference at *p-value* < 0.05

Table S6. The statistical tests of gene expression in honeybee pupae

Caspaes3-like						
**Time** (h)		Kruskal-Wallis	Pairwise Comparisons of treatment (Significance values have been adjusted by the Bonferroni correction for multiple tests)			
	H	***p*-value**	**Treatment**		H	***p*-value**
0	6.867	0.143	-		-	-
6	2.329	0.675	-		-	-
12	5.167	0.271	-		-	-
24	2.067	0.723	-		-	-
48	7.433	0.115	-		-	-
72	1.767	0.779	-		-	-
96	10.600	**0.031***	Control	PBS	1.000	0.784
			Control	*E.coli*	-7.667	**0.036***
			Control	DWV10^{4}	-7.333	**0.044***
			Control	DWV10^{7}	-7.667	**0.036***
			PBS	*E.coli*	-8.667	**0.018***
			PBS	DWV10^{4}	-6.667	0.068
			PBS	DWV10^{7}	-8.667	**0.018***
			E.coli	DWV10^{4}	2.000	0.584
			E.coli	DWV10^{7}	0.000	1.00
			DWV10^{4}	DWV10^{7}	-2.000	0.584

Caspaes8-like						
**Time** (h)		Kruskal-Wallis	Pairwise Comparisons of treatment (Significance values have been adjusted by the Bonferroni correction for multiple tests)			
	H	***p*-value**	**Treatment**		H	***p*-value**
0	8.600	0.072	-			-
6	2.267	0.687	-			-
12	3.298	0.509	-			-
24	1.369	0.850	-			-
48	2.267	0.687	-			-
72	2.296	0.682	-			-
96	10.586	**0.032***	Control	PBS	-1.667	0.648
			Control	*E.coli*	-7.333	**0.044***
			Control	DWV10^{4}	-8.667	**0.018***
			Control	DWV10^{7}	-9.000	**0.014***
			PBS	*E.coli*	-5.667	0.120
			PBS	DWV10^{4}	-7.000	0.055
			PBS	DWV10^{7}	-7.333	**0.044***
			E.coli	DWV10^{4}	-1.333	0.715
			E.coli	DWV10^{7}	-1.667	0.648
			DWV10^{4}	DWV10^{7}	-0.333	0.927

\- = not a statistical different test

* = the statistically significant difference at *p-value* < 0.05

Table S6. The statistical tests of gene expression in honeybee pupae

Caspaes9-like						
**Time** (h)	Kruskal-Wallis		Pairwise Comparisons of treatment (Significance values have been adjusted by the Bonferroni correction for multiple tests)			
	H	***p*-value**	Treatment		H	***p*-value**
0	8.741	0.068	-			-
6	0.667	0.955	-			-
12	3.933	0.415	-			-
24	0.900	0.925	-			-
48	2.433	0.657	-			-
72	5.986	0.200	-			-
96	11.200	**0.024***	Control	PBS	-1.333	0.715
			Control	*E.coli*	-7.333	**0.044***
			Control	DWV10^4	-8.333	**0.022***
			Control	DWV10^7	-10.000	**0.006***
			PBS	*E.coli*	-4.000	0.273
			PBS	DWV10^4	-7.000	0.055
			PBS	DWV10^7	-8.667	**0.018***
			E.coli	DWV10^4	-3.000	0.411
			E.coli	DWV10^7	-4.667	0.201
			DWV10^4	DWV10^7	-1.667	0.648

- = not a statistical different test

* = the statistically significant difference at *p-value* < 0.05
